# Draft genome sequence of *Galbibacter* sp. PAP.153, isolated from a marine sponge in Papua, Indonesia

**DOI:** 10.1128/mra.01297-23

**Published:** 2024-04-30

**Authors:** Riyanti ‌, Celine M. Zumkeller, Marius Spohn, Sanja Mihajlovic, Oliver Schwengers, Alexander Goesmann, Riviani Riviani, Maria Dyah Nur Meinita, Dewi Wisudyanti Budi Hastuti, Isnaini Prihatiningsih, Rose Dewi, Till F. Schäberle

**Affiliations:** 1 Faculty of Fisheries and Marine Science, Jenderal Soedirman University, Purwokerto, Indonesia; 2 Branch for Bioresources, Fraunhofer Institute for Molecular Biology and Applied Ecology (IME), Giessen, Germany; 3 Institute for Insect Biotechnology, Justus Liebig University Giessen, Giessen, Germany; 4 Bioinformatics and Systems Biology, Justus Liebig University Giessen, Giessen, Germany; 5 German Center for Infection Research (DZIF), Partner Site Giessen-Marburg-Langen, Braunschweig, Germany; Montana State University, Bozeman, Montana, USA

**Keywords:** Bacteroidetes, *Galbibacter*, natural products, flexirubin, Indonesia

## Abstract

*Galbibacter* sp. PAP.153 was isolated from a marine sponge. Here, we report its 4.12 Mbp draft genome sequence and rate its specialized metabolite production capacity with specific focus on the chemotaxonomic marker flexirubin.

## ANNOUNCEMENT

The phylum Bacteroidetes is a promising bioresource for discovering novel natural products (1–3) and is abundant in ocean ecosystems (4). The strain PAP.153 was isolated from Cenderawasih Bay National Park, Papua, Indonesia (2°23′6.8″S134°57′53.5″E) and deposited as EXT111903 in the Fraunhofer strain collection (5).

EXT111903 was isolated from sponge material retrieved with a diving knife while wearing latex gloves. The sample was placed in a sterile bag, carried to the surface, stored at ambient seawater temperature, and processed within 2 hours. The sponge material was washed with sterile seawater, sliced using a sterile scalpel, and plated on artificial seawater agar plates (1.5% agar, 0.01% KBr, 2.3% NaCl, 1.1% MgCl_2_·6H_2_O, 0.1% CaCl_2_·2H_2_O, 0.1% KCl, 0.004% SrCl_2_·6 H_2_O, 0.4% Na_2_SO_4_, 0.02% NaHCO_3_, and 0.003% H_3_BO_3_ in H_2_O), including *Escherichia coli* prey and cycloheximide. Distinct colonies were isolated, and sequencing samples were prepared by cultivating strains aerobically for 24 h at 30°C in marine broth (product number CP73.1; Carl Roth GmbH). EXT111903, distinguished by its yellow phenotype, was chosen because it was assigned to the Bacteroidetes phylum.

Cells were pelleted and resuspended in ATL buffer (Qiagen) containing RNase A. ZR BashingBead Lysis Tubes (Zymo Research) were used for cell disruption. DNA was isolated using QIAmp 96 DNA QIAcube HT kits with the addition of proteinase K (Qiagen). Libraries for short-read sequencing were prepared using the Illumina DNA Prep Tagmentation kit with 500 ng of DNA input and five cycles of indexing PCR. Library quality was evaluated (Agilent 2100 Bioanalyzer) and sequenced at an Illumina NovaSeq using a NovaSeq 6000 SP v1 sequencing kit with 2 × 150 bp read length and a depth of 4.0–5.0 Mio reads per sample. The sequencing was demultiplexed (Illumina bcl2fastq, v2.19.0.316), quality checked (Fastq, v0.20.1), and visualized (MultiQC, v1.7). Paired-end reads were quality-filtered [Fastp (6) v0.20.1], assembled [Unicycler (7) v0.4.8], annotated [PGAP (8)], and quality-checked [CheckM (9) v1.0.18]. The genome comprising 4.12 Mbp in 105 contigs (coverage: 351-fold and *N*_50_: 145,281 bp) with a GC content of 38.6% revealed completeness of 100% and contamination of 0.47%. It encodes 3,664 protein-coding genes, 41 tRNAs, 1 tmRNA, 3 rRNAs, and 7 ncRNAs. The Type Strain Genome Server (10) identified *Galbibacter pacificus* CMA-7T (GCF_029603155.1) as the closest related type strain. High digital DNA-DNA hybridization values of 77.6% (d0), 83.3% (d4), and 81.5% (d6) and an ANI (11) of 98.02% surpassing the species delineation threshold confirm PAP153’s association with the species of *G. pacificus*.

**Fig 1:**
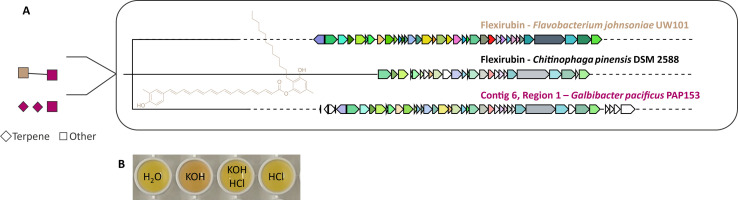
BiG-SCAPE network (A, left) of the strain PAP153 (magenta). One BGC shares similarity (similarity index: 0.430) to the reference BGC of flexirubin from *Flavobacterium johnsoniae* UW101 (brown square). Known flexirubin BGCs were aligned using Corason (A, right) (16) with the BGC detected in PAP153, indeed indicating the presence of complete flexirubin BGC. The color change (B) upon adding 20% KOH solution to crude methanolic extract generated from 2-day cultures of PAP153 in liquid NB further indicates the production of flexirubin. This color change is reversible upon the addition of HCl. The water and HCl controls remain colorless.

AntiSMASH v6.0 (12) was used to predict biosynthetic gene clusters (BGCs). *G. pacificus* PAP.153 contains four BGCs, two terpene-type and two aryl polyene-type clusters. BiG-SCAPE analysis including MiBIG reference BGCs (13) (cutoff: 0.6) grouped one BGC with the flexirubin BGC, a chemotaxonomic marker for Bacteroidetes (14) (Fig. 1A). Flexirubin production in NB medium (0.5% peptone and 0.3% malt extract) at 28°C for 2 days was confirmed by adding 20% KOH (potassium hydroxide, CAS: 1310-58-3) solution to their methanolic crude extracts (15). A flexirubin-indicative KOH-induced color change was detected and reversible by adding HCl (hydrochloric acid, CAS: 7647-01-0) (Fig. 1B).

## Data Availability

The whole-genome shotgun project has been deposited at DDBJ/ENA/GenBank under BioProject PRJNA1049751 with accession number SAMN38709845. Raw data can be obtained from the sequence read archive (SRX23001679). The draft genome sequence has been deposited in GenBank under the accession number JAZEUN000000000.
